# Hierarchical Modeling of the Liver Vascular System

**DOI:** 10.3389/fphys.2021.733165

**Published:** 2021-11-16

**Authors:** Aimee M. Torres Rojas, Sylvie Lorente, Mathieu Hautefeuille, Aczel Sanchez-Cedillo

**Affiliations:** ^1^Department of Mechanical Engineering, Villanova University, Villanova, PA, United States; ^2^Departamento de Física, Facultad de Ciencias, Universidad Nacional Autónoma de México, Ciudad de México, Mexico; ^3^Laboratorio de Trasplantes, Centro Médico Nacional 20 de Noviembre, Instituto de Seguridad y Servicios Sociales de los Trabajadores del Estado (ISSSTE), Ciudad de México, Mexico

**Keywords:** liver circulatory system, constructal design, small-for-size syndrome, hepatectomy, liver hemodynamic parameters

## Abstract

The liver plays a key role in the metabolic homeostasis of the whole organism. To carry out its functions, it is endowed with a peculiar circulatory system, made of three main dendritic flow structures and lobules. Understanding the vascular anatomy of the liver is clinically relevant since various liver pathologies are related to vascular disorders. Here, we develop a novel liver circulation model with a deterministic architecture based on the constructal law of design over the entire scale range (from macrocirculation to microcirculation). In this framework, the liver vascular structure is a combination of superimposed tree-shaped networks and porous system, where the main geometrical features of the dendritic fluid networks and the permeability of the porous medium, are defined from the constructal viewpoint. With this model, we are able to emulate physiological scenarios and to predict changes in blood pressure and flow rates throughout the hepatic vasculature due to resection or thrombosis in certain portions of the organ, simulated as deliberate blockages in the blood supply to these sections. This work sheds light on the critical impact of the vascular network on mechanics-related processes occurring in hepatic diseases, healing and regeneration that involve blood flow redistribution and are at the core of liver resilience.

## Introduction

The liver is one of the largest and essential organs in the body. As such it filters all the blood, with about 75% being deoxygenated blood from the portal vein, while the rest is fully oxygenated blood coming from the hepatic artery. The portal vein and the hepatic artery split into smaller and smaller branches in a dendritic fashion, all the way down to the lobules, the small constitutive units of the liver. There, a triad made of the smallest branches of the portal vein, the hepatic artery and the bile duct connects the vertices of the hexagonal shaped cross section of the lobules. The blood is then distributed into the sinusoids, small, entangled vessels carrying the blood toward the central vein of each lobule. The central veins connect to each other in, again, a tree-shaped way, and reconstruct a third network that drives the blood out of the liver into the inferior vena cava (IVC).

Understanding the vascular anatomy of the liver is clinically relevant because various liver pathologies are related to vascular alterations. One of them is the small-for-size syndrome (SFSS), resulting from major hepatectomy or liver transplantation from small donors or partial grafts. In this syndrome, the liver mass is insufficient to maintain its proper function. Portal hypertension and flow disturbances are maybe the most important injury mechanisms responsible for endothelial damage in the sinusoids. The evolution of the SFSS is not only related to the volume of the remaining organ but also by the parameters of the hepatic circulation, especially by the blood flow in the portal vein (Golriz et al., 2016).

Among the various works on the vascular system of the liver, most of them usually address a part of the hepatic vasculature and / or study the liver of animals such as the mice, dogs, or ferrets (Puhl et al., 2003; Van der Plaats et al., 2004; Debbaut et al., 2012a; Nishii et al., 2016). Some models of the human hepatic circulatory system have also been proposed using different approaches of the vasculature depending on the study undertaken. In White et al. (2016) a 3D model of the portal venous network and the hepatic venous network is used to analyze the diffusion of drugs in the liver. The model does not account for the arterial network. In Ma et al. (2019) a 3D structure of the first vessels of each hepatic vascular network is proposed. In this work, the flow and pressure of the blood in the largest vessels of each network is analyzed in detail, but the hepatic circulatory system is not studied in an integrated way. In Debbaut et al. (2011) and Wang et al. (2017) the analysis of the complete hepatic circulatory system is proposed by means of an electrical analogy. The lack of studies that integrate the different parts of the hepatic vasculature, with their own structural and flow characteristics based on first principles, from macrocirculation to microcirculation, appears clearly from this analysis of the relevant literature.

An integrated model of the circulatory system of the human liver was recently proposed (Lorente et al., 2020). It consists in the superposition of the three hepatic vascular networks, considered as tree-like fluidic networks, combined with lobules that form the capillary bed as a porous medium. The work showed that the main geometric characteristics of the networks and the permeability of the lobules can be defined from the constructal law point of view. According to the constructal law of evolutionary design (Bejan and Lorente, 2008, 2013), given enough freedom, flow systems evolve in time to facilitate access to the currents that flow through them.

The HA network experiences pulsatile flow as the entire arterial system. One way to account for it is to model the HA network flow by means of Fourier analysis as presented in Flores et al. (2010). Nevertheless, as explained in Torres et al. (2015), the contribution of the imaginary part of the impedance is often negligible in the electrical analogy of low frequencies pulsating flows in rigid networks (such as the human heart rate). In this case, the impedance is practically indistinct from that of the non-pulsatile flow. Therefore, we consider here that a direct proportionality exists between the pressure changes and the mass flow rate. In the PV and HV networks, the laminar blood flow obeys Poiseuille flow.

We develop a deterministic model of the human liver vasculature based on the work of Lorente et al. (2020). We propose a theoretical framework to find the rules for the construction of the vascular networks of our model. Using data available in the literature on hemodynamic and geometrical parameters in the liver, we obtain the construction rules that allow us to reproduce the physiological conditions reported for a healthy liver. We use the present model to simulate the SFSS resulting from major hepatectomy.

## Background

**FIGURE 1:**
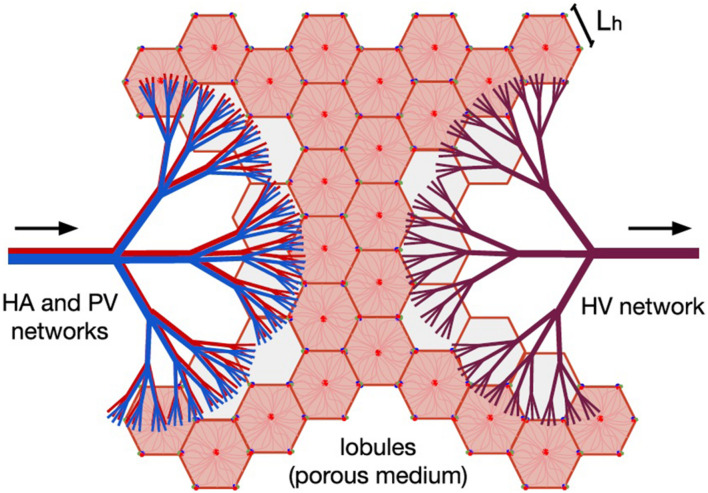
Model of human liver circulation, adapted from Lorente et al. (2020). This model considers the hepatic vascular networks HA (in red), PV (in blue), and HV (in purple) as dendritic fluid networks, and each lobule as a component of a porous medium. Arrows indicate the direction of blood flow. Blood enters the liver through the HA and PV networks, then it is distributed among the existing lobules, and finally it is collected by the HV network responsible for draining the organ.

The entire hepatic circulatory system is modeled as two tree-shaped structures –hepatic artery (HA) and portal vein (PV) networks– supplying the blood to a porous medium –the lobules–, where it mixes before leaving the liver through a single vasculature –the hepatic vein (HV) network–. In Figure 1, the two hepatic blood supply vasculatures (the HA and PV networks) are shown in red and blue, respectively, the lobules (the hepatic functional units shaped like hexagonal prisms) are represented by pink hexagons; and the blood outlet network (the HV network) is shown in purple.

### Tree-Shaped Networks

The HA, PV and HV structures are considered as tree-like networks of rigid cylindrical vessels that split into *n* new vessels –identical to each other– from one generation level *i* to another. In an analysis of experimental data available in the published literature (Debbaut et al., 2011, 2014), Lorente et al. (2020) concluded that, on an average, *n* = 2.76, *n* = 2.8, and *n* = 3.22 for the HA, PV and HV networks, respectively. A more extensive discussion on this analysis is found in section “Discussion” of this paper. A third reference (Ma et al., 2019) combined to the two first ones allowed to link the splitting level to the diameter ratio of a mother branch *i* to its daughter vessels *i+1*. By invoking the constructal law, Lorente et al. (2020) show that the existing diameter ratio *d*_*i* + 1_/*d*_*i*_ is the one leading to minimum friction losses –and therefore minimum pumping power– for a constant fluid volume, in laminar flow. Note that this result is similar to the one obtained with the Hess-Murray’s law which searches for minimum fluid volume. Choosing an integer value for *n*(*n* = 3), the authors noted that the available measurements of the geometrical characteristics agree with *d*_*i* + 1_/*d*_*i*_ = 3^−1/3^, and found that the vessels lengths ratio also follows a similar behavior with *l*_*i* + 1_/*l*_*i*_ = 3^−1/3^.

### Lobules as a Porous Medium

The networks that supply blood to the liver (HA and PV) lead to the hepatic lobules through their smallest vessels (the hepatic arterioles and the portal venules). The classical shape to represent the lobules is an hexagonal prism, in which a portal triad (made of an hepatic arteriole, a portal venule and a bile duct) is located at each of the vertices of the hexagon, and a central vein (the smallest vessels of the HV network) is in the center. The blood flow through the lobule goes from the portal triads to the central vein by bathing entirely the entangled sinusoids network that constitutes the lobules. Under this approach, it can be said that the number of lobules and central veins in the liver is the same.

Lorente et al. (2020) showed that if the transport of blood through the lobule is described as a flow through a functionalized porous system, its corresponding permeability is in the range of data available in the literature.

With the assumption that each portal triad feeds equally three lobules, the mass flow rate through one lobule is therefore ${\dot{m}}_{l\,o\,b\,u\,l\,e}=6\,\rho \,U\,L_{h}\,t$ (Lorente et al., 2020), where *U* is the flow average velocity, *L_h* is the side of the lobule hexagonal cross section, and *t* is its thickness. The average velocity is related to the permeability *K* through Darcy’s law, leading to


(1) $${\dot{\text{m}}}_{\mathrm{lobule}}=\frac{\text{K}}{\nu }\,6\,\text{t}\,△\,{\text{p}}_{\mathrm{lobule}},$$

Where ν is the kinematic viscosity of the blood, and △*p*_*lobule*_ is the pressure loss from the inlet (triad) to the outlet (central vein).

## Vascular Model of a Healthy Liver

The constructal ratios for the vessels diameters and lengths are purely deterministic and serve as an indicator of what the ideal fluid network should be, should it have to be engineered. Next, to account for the actual blood dendritic networks of the liver, we rely both on the rules presented previously and on boundary conditions obtained from experimental data, namely for each network: inlet and outlet pressure, mass flow rate, and volume of the network. From the constructal approach we write that a network is made of the regular generation of vessels in such a way that the radius ratio is constant, and the length ratio is also constant.

As observed in real life, the branching pattern of human livers is not homogeneous. Bifurcations, trifurcations, and even monopodial side branches have been reported. Here, we consider tree-like networks with a single branching pattern, so we use the following theoretical framework for the cases of purely trifurcated networks (*n=3*) and purely bifurcated networks (*n=2*).

Assuming a pressure drop (△*p*_*i*_) in the vessels of each level *i* of the network, the total pressure drop in the network (△*p*) is given by Poiseuille’s law


(2) $$△\,\text{p}=\sum_{i=0}^{k}△\,{\text{p}}_{i}=\frac{8\,\nu }{\pi }\,\sum_{i=0}^{k}{\dot{\text{m}}}_{i}\,\frac{{\text{l}}_{i}}{{\text{r}}_{i}^{4}},$$

where the zero level (*i=0*) is for the first vessel of the network; *k* is the maximum generation level; ${\dot{m}}_{i}$, *l_i* and *r_i* are, respectively, the mass flow rate, the length, and the radius of vessels of each level *i*.

From mass conservation, the inlet mass flow rate (${\dot{m}}_{0}$) is related to the mass flow rate in each vessel of level *i* (${\dot{m}}_{i}$) as:


(3) $${\dot{\text{m}}}_{0}={\text{n}}^{i}\,{\dot{\text{m}}}_{i},$$

We name *B_i* to the ratio $l_{i}/r_{i}^{4}$ in Eq. (2). Assuming a constant ratio *c* = *B*_*i*_/*B*_*i* + 1_, we have the recurrence relation *B*_*i*_ = *c*^−*i*^*B*_0_, where $B_{0}=l_{0}/r_{0}^{4}$, *l_0* and *r_0* are the length and radius of the first vessel in the network. Using this recurrence relation and Eq. (3), we can write Eq. (2) in terms of ${\dot{m}}_{0}$ and *B_0*.

Finally, using $\sum_{j=0}^{N-1}a^{j}=\frac{a^{N}-1}{a-1}$, the total pressure drop in the network is:


(4) $$△\,\text{p}=\frac{8\,\nu }{\pi }\,\dot{{\text{m}}_{0}}\,{\text{B}}_{0}\,\left[\frac{{\left(1/\text{nc}\right)}^{k+1}-1}{\left(1/\text{nc}\right)-1}\right].$$

The total volume of the network is given by:


(5) $$\text{V}=\pi \,\sum_{i=0}^{k}{\text{n}}^{i}\,{\text{r}}_{i}^{2}\,{\text{l}}_{i}.$$

Because the radius and length ratios are constant, the ratio $b=r_{i}^{2}\,l_{i}/r_{i+1}^{2}\,l_{i+1}$ is also constant. Hence, $r_{i}^{2}\,l_{i}=b^{-i}\,r_{0}^{2}\,l_{0}$, and Eq. (5) is rewritten as,$V=\pi \,r_{0}^{2}\,l_{0}\,\sum_{i=0}^{k}{\left(n/b\right)}^{i}$. Finally, we have:


(6) $$\text{V}=\pi \,{\text{r}}_{0}^{2}\,{\text{l}}_{0}\,\left[\frac{{\left(\text{n}/\text{b}\right)}^{k+1}-1}{\left(\text{n}/\text{b}\right)-1}\right].$$

To relate the constants of proportionality *b* and *c*, we start from


(7) $$\text{b}=\left({\text{r}}_{i}^{2}\,{\text{l}}_{i}\right)/\left({\text{r}}_{i+1}^{2}\,{\text{l}}_{i+1}\right)$$

and


(8) $$\text{c}=\left(\frac{{\text{l}}_{i}}{{\text{r}}_{i}^{4}}\right)/\left(\frac{{\text{l}}_{i+1}}{{\text{r}}_{i+1}^{4}}\right).$$

Combining Eqs. (7) and (8) together, we obtain


(9) $$\text{b}={\text{c}}^{-1/2}\,{\left(\frac{{\text{l}}_{i}}{{\text{l}}_{i+1}}\right)}^{3/2}.$$

**FIGURE 2:**
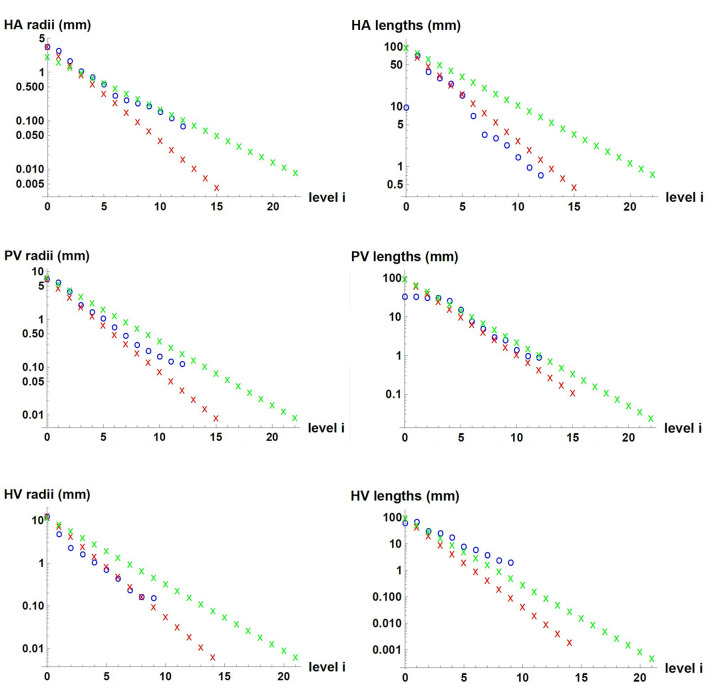
Dimensions of the vessels in the three hepatic vascular networks. From top to bottom, the values for HA, PV and HV networks are presented. The radii (left hand side) and lengths (right hand side) of the vessels of each level *i* of the networks are shown. The red and green crosses represent the values obtained with our model for *n=3* and *n=2*, respectively, and the blue circles, the available experimental measurements (Debbaut et al., 2011, 2014).

We used measured data and estimates found in the literature for △*p*, $\dot{m_{0}}$, *r_0*, *l_0* and *V* of each of the three vascular networks of the liver. The parameters used for each network are provided in Table 1 for *n=3*, and in Supplementary Table 1 for *n=2*. Unknown is the maximum generation level of each network, *k*. Therefore, we consider one value of *k* at a time and determine *b*, *c*, *l*_*i*_/*l*_*i* + 1_ and finally *r*_*i*_/*r*_*i* + 1_. We tested values of *k* that would allow us to obtain radii of the smallest vessels of the networks within the range reported in the literature (Crawford et al., 1998). This process was carried out with the three hepatic vascular networks until obtaining a ratio *r*_*i*_/*r*_*i* + 1_ as close as possible to the experimental data (Debbaut et al., 2011, 2014), and finally the value of *l*_*i*_/*l*_*i* + 1_. Table 2 shows the results obtained for the HA, PV and HV networks, when *n=3*, and Supplementary Table 2 provides the equivalent results when *n=2*. Figure 2 shows the dimensions of the vessels of the three hepatic vascular networks (HA, PV and HV networks, from top to bottom) for bi- and tri- furcated cases. The radii (left hand side) and lengths (right hand side) of the vessels of each level *i* of the network are presented. The red and green crosses are the values obtained with our model for *n=3* and *n=2*, respectively, and the blue circles represent the available experimental measurements (Debbaut et al., 2011, 2014).

**TABLE 1:** Parameters used for a healthy liver networks in the model.

	HA network	PV network	HV network	References
V (mL)	25	75	100	There are 500 g of blood in a 2,000 g liver (Radu-Ionita et al., 2020). This means ∼ 500 mL of blood. Rough estimation suggests ∼ 40% of the hepatic blood is held in the vascular networks (Eipel et al., 2010): 200 mL. We assumed that the anatomical volume of each network is proportional to the blood flow in each one.
*r_0* (mm)	3.5	7.3	13	Debbaut et al., 2011
*l_0* (mm)	100	100	100	Estimated from the data reported in Debbaut et al. (2011) and Rajput et al. (2014).
${\dot{m}}_{0}$ (Kg/s)	4.4×10^−3^	13.5×10^−3^	17.9×10^−3^	McAvoy et al., 2016
*p*_in_ (mmHg)	91*	10**	4***	*(Hadaegh et al., 2012) **(Lebrec et al., 1997) ***Considering a pressure drop of 1 mmHg in the lobules, as reported in Bosch et al. (2009).
*p*_out_ (mmHg)	5	5	3	Estimated from Lautt (1977) and Lebrec et al. (1997).

**TABLE 2:** Construction parameters of the networks.

				% Deviation with respect to the constructal value (3^1/3^)		% Deviation with respect to the measured data
Network	*k*	*l*_*i*_/*l*_*i* + 1_	*r*_*i*_/*r*_*i* + 1_	for *l*_*i*_/*l*_*i* + 1_	for *r*_*i*_/*r*_*i* + 1_	for *r*_*i*_/*r*_*i* + 1_
HA	15	1.43	1.56	0.7	8.2	13.0
PV	15	1.57	1.56	8.9	8.4	10.2
HV	14	2.16	1.72	49.9	19.2	2.4

We consider each hepatic lobule that makes up the capillary bed as a porous medium. We define the number of lobules in our model identical to the number of central veins (the smallest vessels of the HV network), that is, ∼4.8×10^6^ for *n=3* (see the details in Supplementary Table 3 for *n=2*). This number of lobules agrees in magnitude with estimates reported in the literature (1.5 million; Rezania et al., 2016). We calculate the total volume of lobules as the subtraction of the total volume of the liver (1,115 Ml; Mise et al., 2014) minus the volume of the three vascular networks.

Knowing the volume of one lobule, its thickness *t* can be estimated by considering the hexagonal shape of its cross section: $t=\frac{2\,V_{l\,o\,b\,u\,l\,e}}{3\,\sqrt{3}\,L_{h}^{2}}$, where *V*_*lobule*_ is the volume of a lobule. *L_h* is given by data reported in the literature (Bonfiglio et al., 2010; Rezania et al., 2013; Debbaut et al., 2014).

Finally, from Eq. (1) we obtain a lobule permeability that matches the pressure drop estimation of ∼1 mmHg as reported in the literature (Bosch et al., 2009). We have *K* = 1.32×10^−14^ m^2^, a value in the range of lobule permeabilities published in different articles: *K* = 4.8×10^−9^−1.5×10^−14^ m^2^ (Debbaut et al., 2012b; White et al., 2016). Table 3 summarizes the parameters used for a healthy liver lobule in our model for *n=3* (Supplementary Table 3 for *n=2*).

**TABLE 3:** Parameters used for the healthy liver lobule.

Number of lobules	4782969
*V*_*lobule*_ (mL)	1.9×10^−4^
*L_h* (mm)	0.25
*t* (mm)	1.2
*K* (m^2^)	1.32×10^−14^
${\dot{m}}_{l\,o\,b\,u\,l\,e}$ (Kg/s)	3.7×10^−9^
*p*_in_ (mmHg)	5
*p*_out_ (mmHg)	4

In sum, the vascular model presented here reproduces the flows and pressures in a liver in healthy conditions.

## Small-For-Size Syndrome Modeled by Liver Resection (Hepatectomy)

### Resection Model

We simulate hepatectomies by “removing” different portions of the healthy liver, which we call resection percentages. Our model of a healthy liver is made of the three hepatic vascular networks and of the porous medium constituted by the lobules. To “remove” a portion of the liver, we obstruct certain number of vessels (by making their cross-sectional areas tend to zero) in all three vascular networks and reduce the number of lobules in the same proportion.

To resect different portions of the liver, we decided to be guided by the vasculature. In theoretical and experimental animal models, hepatectomy is usually done by resecting one or more lobes of liver because these are well differentiated in them, for example, in rats or pigs (Smyrniotis et al., 2002; Debbaut et al., 2012a; Audebert et al., 2017). The human liver is also made up of lobes, but these are not well differentiated, so in practice one would block the blood flow from one of the main branches of PV network that supply the liver and observe the location of the tissue that begins to change color due to the lack of blood irrigation and then cut in those limits.

We consider that, in a liver resection, it is possible to access (and therefore to close) the largest vessels of the networks: level *i=1* and level *i=2*. The following results correspond to the case *n=3*. The equivalent results for *n=2* are given in Supplementary Material. The closure of the different vessels of these two levels results in eight possible resection schemes (specified in Table 4). Therefore, the vessels indicated in each resection scheme are “resected” (closed), so that all the vessels that are born from them will also be without flow.

**TABLE 4:** Liver resection schemes considered.

Vessels “removed” in the three hepatic networks by making their cross-sectional areas tend to zero.	% of resection (% of vessels “removed”s = % of lobules “removed”)
All vessels belonging to one of the nine branches of level *i=2*.	11
All vessels belonging to two of the nine branches of level *i=2*.	22
All vessels belonging to one of the three branches of level *i=1*.	33
All vessels belonging to one of the three branches of level *i=1* and all vessels belonging to one of the remaining six branches of level *i=2*.	44
All vessels belonging to one of the three branches of level *i=1* and all vessels belonging to two of the remaining six branches of level *i=2*.	56
All vessels belonging to two of the three branches of level *i=1*.	67
All vessels belonging to two of the three branches of level *i=1* and all vessels belonging to one of the remaining three branches of level *i=2*.	78
All vessels belonging to two of the three branches of level *i=1* and all vessels belonging to two of the remaining three branches of level *i=2*.	89

The percentage of resection in each of these schemes is defined as the percentage of vessels that have been “resected” in the networks (through which there is no longer fluid flow). This percentage is in turn the same percentage of smallest vessels affected and the same percentage of lobules that will not be irrigated.

**FIGURE 3:**
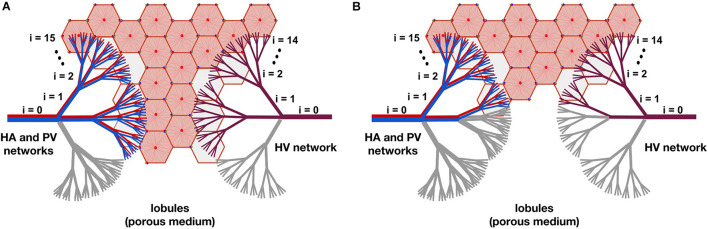
The model of liver resection showing two of the tested resection schemes, 33 and 78% in **(A,B)**, respectively. In **(A)**, all vessels belonging to one of the three branches of level *i=1*, indicated in gray, are “removed” (by making their cross-sectional areas tend to zero). This means “eliminating” 33% of the smallest vessels -vessels at level *i=15* for the HA and PV networks, and vessels at level *i=14* for the HV network-, so 33% of the lobules are also “removed” (reducing the original amount of them). In **(B)**, all vessels belonging to one of the three branches of level *i=1* and to two of the six remaining branches of level *i=2*, indicated in gray, are “removed.” This means the “elimination” of 78% of the smallest vessels of the networks and therefore of the lobules.

In Figure 3, two resection schemes are exemplified, 33 and 78% in (A) and (B), respectively. In Figure 3A, we “removed” all the vessels that belong to one of the three branches of level *i=1*, indicated in gray. This implies the “elimination” of 33% of the smallest vessels of the three networks –vessels at level *i=15* for the HA and PV networks, and vessels at level *i=14* for the HV network–, so we also “eliminate” 33% of the lobules (reducing the original amount of them). In Figure 3B, we “removed” all the vessels belonging to one of the three vessels at level *i=1* and all vessels belonging to two of the remaining six branches of level *i=2*, indicated in gray. This means the “elimination” of 78% of the smallest vessels of the three networks and therefore of the lobules.

### Methodology

We use an electrical analogy for the resistance to the blood flow in each vessel of the networks. The flow resistance in any vessel is $△\,p_{i}/{\dot{m}}_{i}=8\,\nu \,l_{i}/\pi \,r_{i}^{4}$. The assembly of resistances in series and in parallel provides the resistance *R* of a network. We account for the vessels “removed” due to resection by making their cross-sectional areas tend to zero, that is, their resistances tend to infinity. *R* takes different values with the different percentages of resection.

The pressure drop through a network, Δ*p* = *p*_out_−*p*_in_, is given by:


(10) $${\text{p}}_{\text{out}}-{\text{p}}_{\text{in}}=\text{R}\,{\dot{\text{m}}}_{0}.$$

Regarding the lobule, it should be noted that its flow resistance (*ν*/6*Kt*) is the same before and after hepatectomy. The resistance of a single lobule does not change because the structure of the lobule is not modified in the case of resection. Resection does affect the porous medium as a whole: it decreases the number of lobules. Then, after resection, the total blood flow supplied by the HA and PV networks to the lobules is distributed in a smaller amount of them, causing the blood flow in each lobule to increase with the percentage of resection.

**FIGURE 4:**
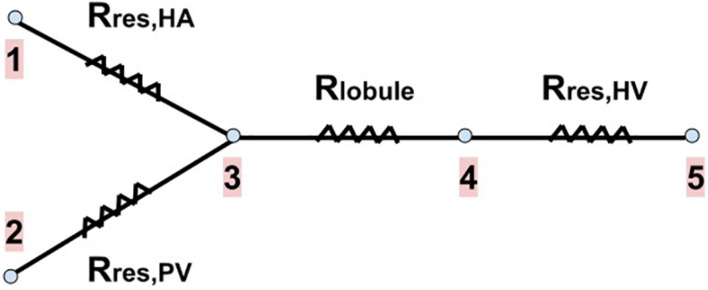
Blood flow pathway with the resistances associated to each part of our vascular liver model. The two branches on the left of the drawing represent the blood supply networks to the liver (the HA and PV networks), the next branch represents a lobule (the blood received from the supply networks is evenly distributed among the lobules), and the last branch represents the HV network (in which blood is collected from all lobules to drain the liver). R_*res*,*HA*_, R_*res*,*PV*_ and R_*res*,*HV*_ are the new resistances of the networks after resection. R_lobule_ is the resistance of a lobule (no change due to resection). Points 1 and 2 represent the inputs of the HA and PV networks, respectively. Point 3 represents the exit of these networks, as well as the entrance to each lobule. Point 4 represents the exit of each lobule and the entrance to the HV network. Point 5 represents the outlet of the HV network, that is, the outlet of the liver (the inferior vena cava, IVC).

We calculate the new resistances of the networks after resection *R*_*res,HA*_, *R*_*res,PV*_ and *R*_*res,HV*_. We consider that both the total mass flow rate of blood entering the liver and its outlet pressure do not change due to the resection, that is, they have the same values as in the healthy liver. The total inlet mass flow rate is given by the flows of the HA and PV networks (${\dot{m}}_{t\,o\,t\,a\,l}={\dot{m}}_{H\,A}+{\dot{m}}_{P\,V}$), and after passing through the lobules, it is collected in the HV network. The outlet of the liver is the IVC, point 5 in Figure 4.

Knowing the pressure at the outlet of the liver (which is also the outlet of the HV network) and *R*_*res,HV*_, we can calculate the new inlet pressure to the HV network due to resection through Eq. (10); this pressure is also the outlet pressure of each lobule (point 4 in Figure 4). Knowing the new lobule outlet pressure and the new mass flow rate received by each lobule, as there are fewer lobules due to resection, we calculate the new lobule inlet pressure (point 3 in Figure 4) using Eq. (1).

The mass flow rate and pressure at the inlets of the HA and PV networks after resection (points 1 and 2 in Figure 4) are related to each other’s through


(11) $${\dot{\text{m}}}_{\mathrm{HA}}+{\dot{\text{m}}}_{\mathrm{PV}}={\dot{\text{m}}}_{\mathrm{total}},$$

(12) $${\text{p}}_{3}-{\text{p}}_{\text{in},\mathrm{HA}}={\text{R}}_{\mathrm{res},\mathrm{HA}}\,{\dot{\text{m}}}_{\mathrm{HA}},$$

and


(13) $${\text{p}}_{3}-{\text{p}}_{\text{in},\mathrm{PV}}={\text{R}}_{\mathrm{res},\mathrm{PV}}\,{\dot{\text{m}}}_{\mathrm{PV}}.$$

The problem closure comes with the choice of a boundary condition. We assume that the HA network inlet pressure is the same before and after resection, and we calculate the new PV network inlet pressure and the new flow distribution.

It is well known that the blood flow and pressure in the PV network increase when the liver is resected. In a review of different studies on hepatectomies in rats, presented in Debbaut et al. (2012a), certain changes were also observed in the total mass flow rate entering the liver, as well as in the blood pressure in the HA network. However, these changes seemed to be insignificant compared to those in the PV network. For this reason, we considered that the total mass flow rate and the inlet pressure of the HA network have the same value as in the healthy liver, regardless the degree of resection.

### Impact of Small-for-Size Syndrome on the Blood Flow and Pressure

We studied different percentages of liver resection considering that the total inlet mass flow rate (${\dot{m}}_{H\,A}$ + ${\dot{m}}_{P\,V}$) and the liver outlet pressure –in the IVC, point 5 in Figure 4– are the same as before the resection. We also consider that the inlet pressure of the HA network is the same as in a healthy liver, and that the inlet pressure of the PV network, as well as the mass flow in each of the supply networks, change due to resection.

**FIGURE 5:**
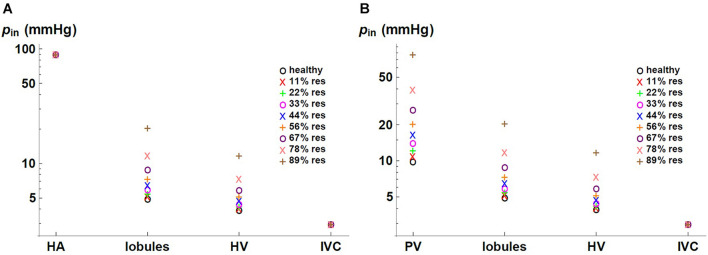
Pressure in **(A)** HA and **(B)** PV pathways for different percentages of liver resection.

**FIGURE 6:**
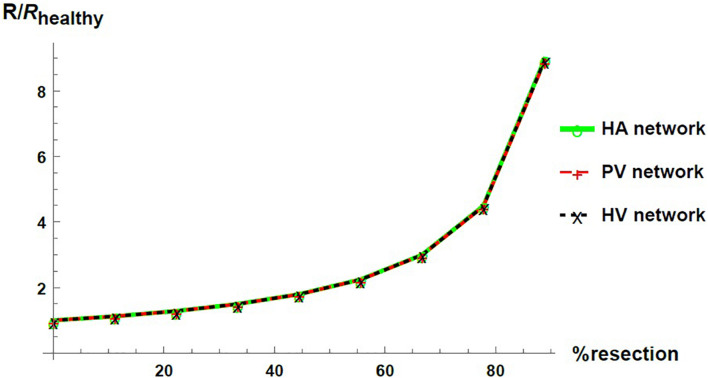
Networks resistance as a function of the resection percentage for HA (green), PV (red) and HV (black) networks. The resistance is normalized by its corresponding value in the healthy liver.

We express the results by considering blood flow pathways from inlets (HA or PV) to the outlet (HV). We present in Figure 5 the pressure at the points of HA and PV pathways for different percentages of resection. As can be seen, the pressure increases in the different places of the hepatic vascular system because of the resection. Furthermore, this increase in pressure appears to be more significant in resections greater than 67%. This observation can be explained by the change in the resistance of the hepatic vascular networks with the percentage of resection. Figure 6 shows the resistance of the HA (in green), PV (in red) and HV (in black) networks as a function of the degree of liver resection. The curves are normalized by their corresponding resistance without resection (*R*_*healthy*_). The curves are superimposed, which means that the resistance of the three vascular networks is affected in the same way by a hepatectomy, but it is evident that the change in resistance is much greater after ∼ 67% resection.

**FIGURE 7:**
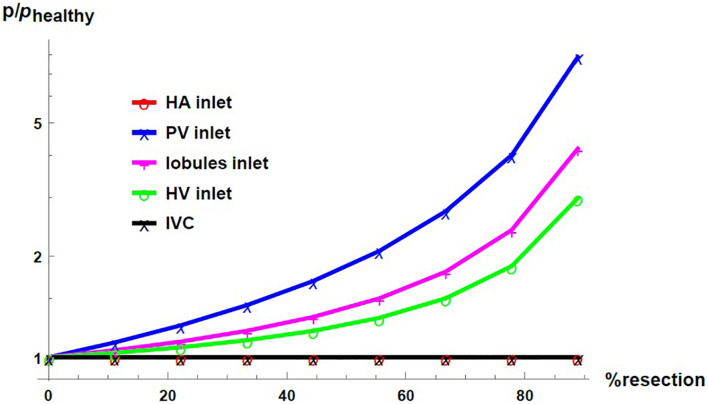
Pressure as a function of the resection percentage in different locations of the liver. The pressure is normalized by its corresponding value in the healthy liver.

Figure 7 shows the pressure as a function of the percentage of resection in different locations of the hepatic vascular system. The pressure is normalized with respect to its corresponding value in the healthy liver, *p*_*healthy*_. As established with the boundary conditions, the pressure at the entrance of the HA network and at the exit of the liver –in the IVC, IVC– do not change due to the resection. At the entrance of the PV network, at the entrance of the lobules and at the entrance of the HV network, the pressure increases considerably when the percentage of resection increases. Such result indicates that the impact caused by resection is different at different locations in the hepatic vasculature. Hepatectomy leads to a greater impact on the pressure the furthest away from the outlet of the organ, that is, the increase in pressure due to resection is greater in the PV network than in the lobules, and it is greater in the lobules than in the HV network.

**FIGURE 8:**
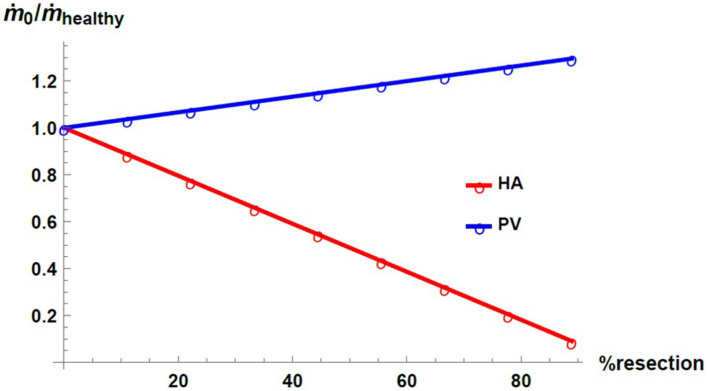
Inlet mass flow rate as a function of resection percentage at the HA network (red) and the PV network (blue). The mass flow rate is normalized by its corresponding value in the healthy liver.

Figure 8 shows the inlet mass flow rate in the HA and PV networks as a function of the percentage of resection (in red and blue, respectively). The mass flow rates are normalized by their respective value in the healthy liver, ${\dot{m}}_{h\,e\,a\,l\,t\,h\,y}$. We can observe that, with the imposed boundary conditions, an effect of liver resection is a redistribution of the inflow to the liver between the two blood supply networks: the mass flow rate in the PV network increases with respect to its value in the healthy liver, while the mass flow rate in the HA network decreases consequently. The differences in the distribution of flow are exacerbated with the degree of resection.

## Discussion

The study of the liver, from a vascular point of view, allows progress in the mechanistic understanding of the impact of vasculature disruptions on hemodynamic parameters (such as flow and pressure). Having a hepatic circulatory model with a deterministic architecture –from the macrocirculation to the microcirculation– is very useful to simulate the vascular alterations underlying the most common scenarios of liver pathology associated with blood flow. For example, liver resection, liver transplantation, portal and hepatic artery thrombosis, and cirrhosis.

The hepatic artery, portal vein and hepatic vein networks are three-dimensional, highly nested, forming an extremely compact structure. In a study of the root branching pattern of the portal network in rats and humans, the most frequently observed branching pattern was a trifurcation for the rat, and a bifurcation for the human (Kogure et al., 1999). However, we have only found a couple of studies in which measurements of the dimensions of the hepatic vessels were carried out at different scales taking samples from the cast of a human liver at the macro, meso and micro levels (Debbaut et al., 2011, 2014). The authors observed branching patterns that include bifurcations, trifurcations, and even monopodial side branches. Beyond reporting the radii and lengths of the vessels, the authors also reported the number of vessels “belonging” to each generation level of the hepatic networks. This assignment was made mainly by following the ramifications of the mother vessels into two or three equal vessels whether it was a bifurcation or a trifurcation. But in the case of monopodial vessels, the assignment was made according to the radius of the vessels: if a certain monopodial vessel had a radius equal to that of the vessels found in generation level x of the network, this vessel was assigned as belonging to level x. In an effort to consider the human hepatic vascular networks, from macrocirculation to microcirculation, in Lorente et al. (2020) the splitting number of the vascular networks of the liver was analyzed from the data reported in Debbaut et al. (2011, 2014). The authors divided the number of vessels assigned to level *i+1* by the number of vessels assigned to level *i* for each of the hepatic networks, and concluded that the HA, PV and HV networks could be considered trifurcated, since this ratio was on average 2.76, 2.80, and 3.22, respectively. The way to assign a generation level in the networks to each measured vessel considered by the authors in Debbaut et al. (2011, 2014) does not necessarily correspond to the real structure of each branching pattern from the macro to the micro scale. Considering that the hepatic vascular networks are trifurcated on an average is one way of taking into account the vessels of the different scales, arranged in a tree-like structure that branches from greater to smaller radius (and length).

Our theoretical framework for modeling tree-like networks considers homogeneous networks (with a single branching pattern). As observed in real life, the branching is not regular and the branching level lies on an average between 2 and 3. We use the proposed theoretical framework for the cases of purely trifurcated networks and purely bifurcated networks. The details of the bifurcated networks case analysis is included in Supplementary Material. It is worth mentioning that the results obtained when considering bifurcated networks follow the trends observed for trifurcated networks, and the conclusions of this work do not change. A model closer to the anatomical complexity of the fluidic networks of the liver could be one that considers a certain proportion of trifurcations and bifurcations.

The objective of the current work was to design vascular networks based on the total volume of the liver, and to understand the impact of resection. Here, the percentages of resection studied are percentages of the total volume of the organ that have been removed, and not dissected lobes. One of the novelties of this work consists in the ability to design/obtain the required network to meet certain constraints of volume, pressure drop and mass flow rate, based on the known dimensions of the initial vessel and knowing the range of dimensions of the smallest vessels to be reached. Considering the total volume of the liver is our first approach to modeling the vasculature of the human liver. One of the next improvements to our circulatory model of the human liver could be to use the theoretical framework developed to obtain the vasculature of each hepatic lobe/portion (left and right) weighted by its characteristic volume and knowing the dimensions of its initial vessel.

Regarding models that study liver hepatectomy, the appropriate boundary conditions are not yet clear. In Debbaut et al. (2012a), partial hepatectomies were studied in a rat model and the dependence of the results with the use of different boundary conditions was shown, as well as with different ways of resecting the same proportion of liver. In Ma et al. (2020) different boundary conditions were also explored in a liver resection model of the human liver, although this study focuses only on the hemodynamic variations in the hepatic artery network. The present study is a further contribution to exploring the appropriate boundary conditions to reproduce and study the main effects of a partial resection or transplantation of a human liver on flow and pressure.

We saw that the impact of liver resection on the flow resistance in the three vascular networks of the liver is the same, as when “resecting” the liver, the same proportion of vessels is eliminated in the three vascular networks. In other words, the change in the resistances of the networks is only due to the structural modification, and this modification is carried out in the same way in the three networks. Yet, by considering that the pressure in the IVC (the outlet vessel of the HV network) is fixed, the pressure change is uneven in the networks. It is considered that the HV network, responsible for draining the blood from the liver, generally does not undergo considerable pressure changes. Therefore, the structural change (in flow resistance) due to the liver resection combined with the fixed outlet pressure and total blood mass flow rate, impact the upstream pressure. The increase in pressure is greater in the PV network than in the lobules, which is in turn greater than in the HV network.

The results agree with the main observations in the SFSS (derived from a major hepatectomy or partial liver transplant). We found that the effect of hepatectomy on portal pressure is much higher for resection percentages greater than ∼ 67%. It has been reported that when an extended hepatectomy is performed, a remnant liver volume smaller than 20–30% of the total liver volume is correlated with the incidence of liver failure and infections (Golriz et al., 2016). We also observe that the blood mass flow rate in the PV network increases with the percentage of resection while the flow in the HA network decreases. In Smyrniotis et al. (2002), an increase in the flow of the portal venous network and a decrease in the flow of the hepatic arterial network were also observed in small-for-size liver transplantations in pigs.

Hepatic arterial blood flow control in healthy subjects is determined by both extrinsic and intrinsic mechanisms. Extrinsic mechanisms mainly result from the action of the neurovegetative system which limits the variability of the body state despite dramatic changes in external conditions. Intrinsic mechanisms are described in terms of local regulation of arterial pressure. Some studies revealed a pressure-dependent autoregulation in local hepatic arterial beds that consist of a myogenic adaptation to changes on the tansmural pressure, leading to a vasoconstrictive response if the arterial pressure rises, attributed to humoral effects. Another intrinsic mechanism is known as the hepatic arterial buffer response (HABR), that consists of compensatory blood changes in response to fluctuations in portal venous flow. Therefore, when the portal blood flow is increased the hepatic artery constricts, and the hepatic artery dilates when the portal flow is reduced. This mechanism is presumably mediated by adenosine, that is cleared by portal circulation, but when it is partially interrupted, adenosine accumulation produces a direct vasodilatory effect on arterial beds (Eipel et al., 2010).

The assumptions to simulate the SFSS allow us to observe: an increase in portal pressure, an increase in portal flow without an increase in effective hepatic flow, and a decrease in arterial flow with a constant hepatic arterial pressure that could be the predominant cause of SFSS. Hence, this model provides another tool to model the pathophysiology of liver failure associated with portal hyperflow, and not only the complications associated with portal hypertension.

Models proposed to simulate hepatectomies, liver transplants or SFSS, require input assumptions that are not defined anywhere and represent an opportunity for exploration in this field of study. Several studies have proposed different inputs (Debbaut et al., 2012a; Ho et al., 2013; Barléon et al., 2018; Ma et al., 2020) in an attempt to find the most useful to reproduce the effects of hepatectomy, liver transplants, and SFSS.

From a mechanical point of view, what parameter remains constant (mass flow rate, pressure, or flow resistance) when the liver undergoes a resection or transplant? It is an open question.

In our opinion, the blood flow is kept constant, because it is in charge of maintaining the tissues alive and transporting the substances required for physiological processes. When there is a disease or a surgical intervention, the liver structure changes and therefore also its resistance. This is why the greatest impact is on the pressure. In the case of the liver, since there are two blood inflows, a redistribution of the total flow could occur, which has been observed in real life.

Recent hypotheses (Golriz et al., 2016) assume that in both major hepatectomy and partial liver transplantation, the hemodynamic parameters of the hepatic circulation are a strong contributor to the development of the SFSS, in addition to the volume of the liver graft or remnant. Furthermore, the smaller new liver volume is believed to generate over-pressure in the portal vein. The same authors indicated that the development of “post-hepatectomy liver failure” may be related to this enhanced portal vein flow. This highlights the importance of continuing to study how to relate hemodynamic parameters of the hepatic circulation to vascular alterations resulting from hepatectomy or partial liver transplantation.

## Conclusion

In this work we developed and validated a deterministic model of the vasculature of the healthy human liver. We proposed a new theoretical framework to find the construction rules of a hepatic circulatory model that allows to obtain the physiological circulatory conditions of a healthy liver. This theoretical framework is fed by measurements and estimates available in the literature on structural and hemodynamic parameters of the liver. The components of the model, such as the dimensions of the vessels that make up the vascular networks, the number of lobules, and the permeability of a lobule, are also within the ranges reported in the literature.

We show the usefulness of our circulatory model of a healthy liver by simulating the SFSS following an extended hepatectomy. We studied its impact on the blood flow and pressure at different location of the vasculature. Our results are consistent with medical observations of hepatectomy, such as increased portal pressure and portal hyperflow.

## Data Availability Statement

The raw data supporting the conclusions of this article will be made available by the authors, upon request.

## Author Contributions

AMTR wrote the code, ran the numerical experiments, and conducted the analysis. SL developed the theory and conducted the analysis. MH provided the experimental data and figures. AS-C brought the medical vision to the work. All authors contributed to the article and approved the submitted version.

## Conflict of Interest

The authors declare that the research was conducted in the absence of any commercial or financial relationships that could be construed as a potential conflict of interest.

## Publisher’s Note

All claims expressed in this article are solely those of the authors and do not necessarily represent those of their affiliated organizations, or those of the publisher, the editors and the reviewers. Any product that may be evaluated in this article, or claim that may be made by its manufacturer, is not guaranteed or endorsed by the publisher.
