# Supplementary material for: Hierarchical Modeling of the Liver Vascular System
Source: Front Physiol. 2021 Nov 16;12:733165. doi: 10.3389/fphys.2021.733165 (PMC8637164; doi:10.3389/fphys.2021.733165)
Supplement: Supplementary file 1 [file Data_Sheet_1.PDF]

Considering bifurcated networks in our human liver vascular model

We apply the theoretical framework developed in this work to build a model of the human liver considering bifurcated networks. We follow the methodology described in section 3 of the article, using a splitting number $n = 2$, corresponding to bifurcations. We present the results obtained in tables and graphs equivalent to the case of trifurcated networks.

As it can be seen, the results obtained when considering bifurcated networks follow the trends observed for trifurcated networks, and the conclusions of the work do not change.

The results related to the construction of the vascular model of the healthy liver are presented in section I. The results related to the small-for size-syndrome modeled as a liver resection are presented in section II.

As explained in section 4.1 of the article, in a liver resection, we consider that it is possible to access (and therefore to close) the largest vessels of the networks: level $i = 1$ and level $i = 2$. The closure of the different vessels of these two levels results in only three possible resection schemes for the case of bifurcated networks (specified in Supplementary Table 4).

1 Healthy Liver

Supplementary Table 1. Parameters used for a healthy liver bifurcated networks in the model.

	HA network	PV network	HV network	References
V (mL)	25	75	100	There are 500 g of blood in a 2000 g liver (Radu-Ionita et al., 2020). This means ~ 500 mL of blood. Rough estimation suggests ~ 40% of the hepatic blood is held in the vascular networks (Eipel et al., 2010): 200 mL. We assumed that the anatomical volume of each network is proportional to the blood flow in each one.
r_0 (mm)	2.15	7.85	12	(Ma et al., 2019)
l_0 (mm)	100	100	100	Estimated from the data reported in Debbaut et al. (2011) and Rajput et al. (2014).
$\dot{m}_0$ (Kg/s)	4.4×10^{-3}	13.5×10^{-3}	17.9×10^{-3}	(McAvoy et al., 2016)

p_{in} (mm Hg)	91*	10**	4***	*(Hadaegh et al., 2012)<br>**(Lebrec et al., 1997)<br>***Considering a pressure drop of 1 mmHg in the lobules, as reported in Bosch et al. (2009).
p_{out} (mm Hg)	5	5	3	Estimated from Lautt (1977) and Lebrec et al. (1997).

Supplementary Table 2. Construction parameters of the bifurcated networks.

				% Deviation with respect to the constructal value ($2^{1/3}$)		% Deviation with respect to the measured data
Network	k	l_i/l_{i+1}	r_i/r_{i+1}	for l_i/l_{i+1}	for r_i/r_{i+1}	for r_i/r_{i+1}
HA	22	1.25	1.28	0.9	1.9	7.1
PV	22	1.46	1.36	15.7	7.9	4.2
HV	21	1.79	1.43	41.7	13.5	14.8

Supplementary Table 3. Parameters used for the healthy liver lobule.

Number of lobules	2097152
V_{lobule} (mL)	4.4×10^{-4}
L_h (mm)	0.25
t (mm)	2.7
K (m^2)	1.32×10^{-14}
$\dot{m}_{lobule}$ (Kg/s)	8.5×10^{-9}
p_{in} (mm Hg)	5
p_{out} (mm Hg)	4

2 Small-for-size syndrome

Supplementary Table 4. Liver resection schemes considered

Vessels "removed" in the three hepatic networks by making their cross-sectional areas tend to zero.	% of resection<br>(% of vessels "removed"<br>= % of lobules "removed")

All vessels belonging to one of the four branches of level $i = 2$.	25
All vessels belonging to one of the two branches of level $i = 1$.	50
All vessels belonging to one of the two branches of level $i = 1$ and all vessels belonging to one of the remaining two branches of level $i = 2$.	75

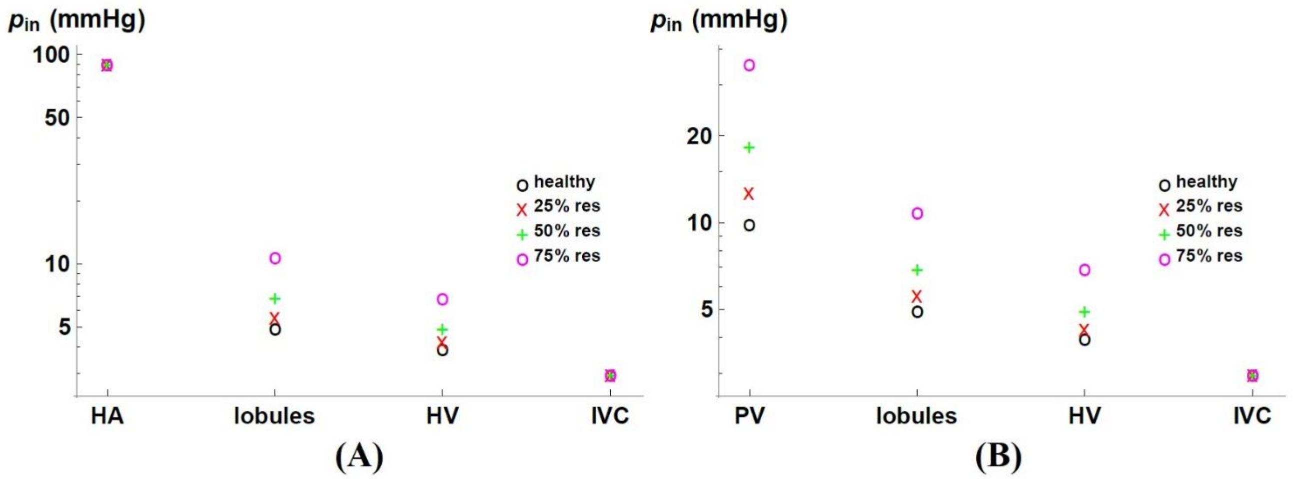

Supplementary Figure 1. Pressure in **(A)** HA and **(B)** PV pathways for different percentages of liver resection.

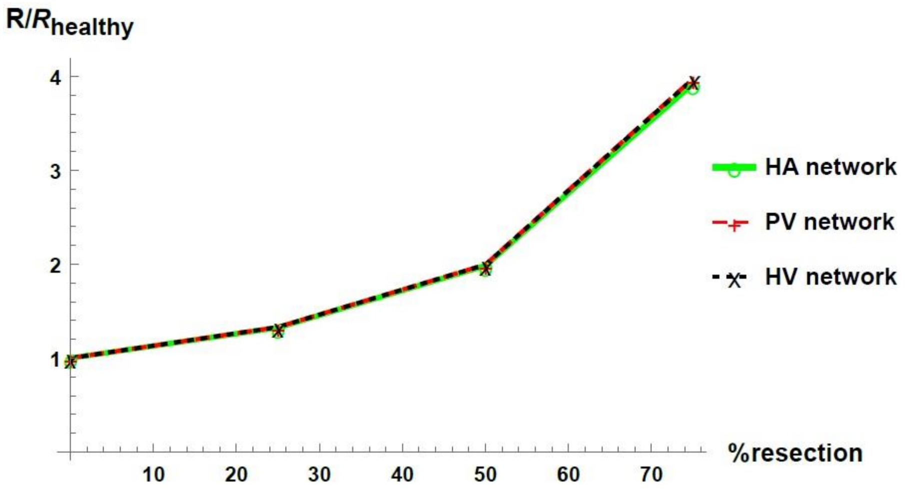

Supplementary Figure 2. Bifurcated networks resistance as a function of the resection percentage for HA (green), PV (red) and HV (black) networks. The resistance is normalized by its corresponding value in the healthy liver.

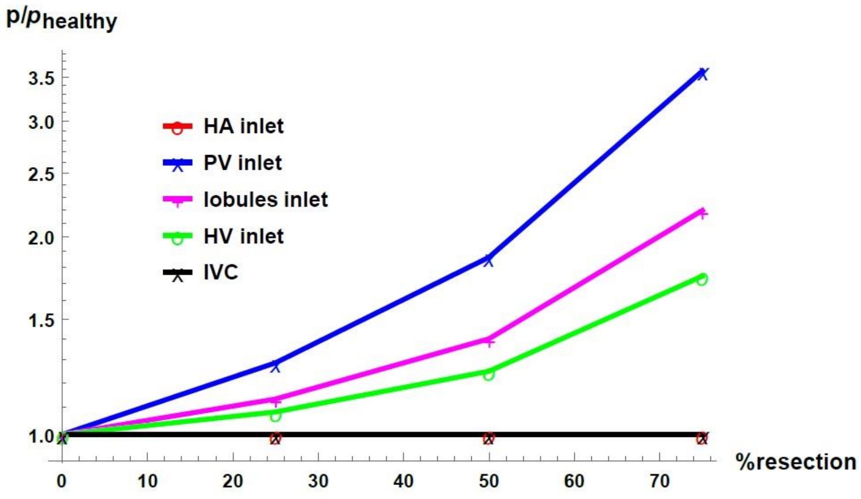

Supplementary Figure 3. Pressure as a function of the resection percentage in different locations of the liver. The pressure is normalized by its corresponding value in the healthy liver.

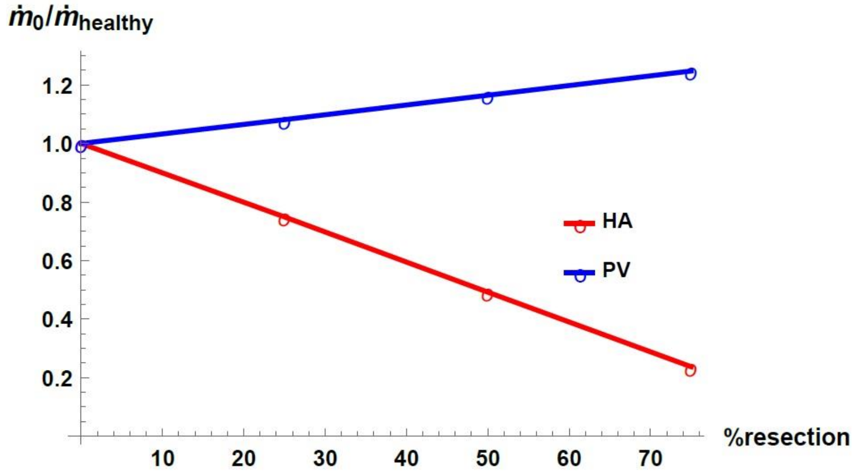

Supplementary Figure 4. Inlet mass flow rate as a function of resection percentage at the HA bifurcated network (red) and the PV bifurcated network (blue). The mass flow rate is normalized by its corresponding value in the healthy liver.
